# Evaluation of the Thuringian Bovine Johne’s Disease Control Program—A Case Study

**DOI:** 10.3390/ani12040493

**Published:** 2022-02-17

**Authors:** Karsten Donat, Esra Einax, Anne Klassen

**Affiliations:** 1 Thüringer Tierseuchenkasse, Anstalt des öffentlichen Rechts, 07745 Jena, Germany; eeinax@thtsk.de; 2 Klinikum Veterinärmedizin, Klinik für Geburtshilfe, Gynäkologie und Andrologie der Groß- und Kleintiere mit Tierärztlicher Ambulanz, Justus-Liebig-Universität Gießen, 35390 Giessen, Germany; aklassen@thtsk.de

**Keywords:** paratuberculosis, voluntary control, participation, calf mortality

## Abstract

**Simple Summary:**

Paratuberculosis, or Johne’s Disease, occurs worldwide, causing a granulomatous enteritis in ruminants. Control programs, which operate mainly on a voluntary basis, have been established in several regions. In Thuringia, a federal state of Germany, a voluntary control program was firstly established in 2003 and revised in 2008 and 2015. This study aimed at evaluating the results achieved in voluntary Johne’s Disease control in Thuringia between 2015 and 2020. By the end of 2020, a total of 67 dairy herds with approximately 33,000 dairy cows and 87 suckler herds keeping approximately 6000 cows of beef breeds were enrolled, representing 35% or 16% of the population, respectively. In affected herds, the program focused on prevalence reduction by hygienic measures to break the infection cycle, annual testing for the detection of animals shedding the infectious agent and culling of these animals. A certification pathway was established based on a testing period of 3 years without positive results, and 58 herds were certified as ‘non-suspect’ with respect to Johne’s Disease. Analyzing the annual calf mortality rate in enrolled dairy farms revealed that herds that had been certified as ‘non-suspect’ regarding Johne’s Disease had a lower calf mortality rate than herds that were not enrolled in the program.

**Abstract:**

The Thuringian Johne’s Disease (JD) Control Program provides a voluntary approach to JD control in Thuringia, a federal state of Germany. The program has three objectives: reduce the level of infection when present; reduce the spread of JD to uninfected herds; and facilitate the certification and protection of herds that are non-suspect with respect to JD. The program offers pathways for the management of affected herds and for certification of herds with continuing negative tests. After the control stage (CS), a certification stage of at least 3 consecutive years with continuing negative results in the annual whole-herd test has to be passed until a herd can be certified as ‘non-suspect’ with respect to JD. This study focused on calf mortality in relation to JD herd status. In a longitudinal study, the association of annual calf mortality rate of a total of 93 dairy herds (13 ‘non-suspect’; 26 in control stage; 54 not enrolled) over 10 consecutive years with JD herd status was investigated using a generalized mixed linear model with repeated measures. Non-suspect herds had a lower calf mortality rate compared with other farms. We conclude that establishing JD control measures lowers the calf mortality rate.

## 1. Introduction

Johne’s Disease (JD), or paratuberculosis, a chronic granulomatous enteritis in cattle, is caused by *Mycobacterium avium* subsp. *paratuberculosis* (MAP). The disease occurs worldwide, mainly in ruminants. Clinical signs are mostly nonspecific, unless intermittent diarrhea, oedema caused by protein deficiency and weight loss despite regular appetite occur frequently. The incubation period lasts up to ten years [1,2]. Decreases in milk yield and carcass value, as well as premature culling, significantly impact the economic performance of affected dairy herds [3,4,5], while studies regarding the economic impact related to infertility and predisposition to other diseases have had varying outcomes [5]. The zoonotic potential of MAP cannot be ruled out, and a metanalysis indicated a significant positive association between MAP exposure and several diseases in humans, including Crohn’s disease [6,7]. In order to reduce human exposure to MAP and the risk of diseases associated with MAP, a mandatory JD Control Program accompanied by modification of pasteurization standards in order to more effectively kill MAP and increased regulation of pasteurization of all dairy products were suggested recently [8].

Because of its impact on animal health, its untreatable nature and the zoonotic aspects, the strategic control of JD has a far-reaching history. Early programs to control JD were set up in France in the 1920s, followed by Great Britain and Iceland in the 1960s [9]. Voluntary control programs have been implemented in several developed countries, e.g., in Australia (1996), South Africa (1997), Canada (2007), New Zealand (2009), the United Kingdom (1998), Spain (2004), Denmark (2006), Belgium (2006), some regions of Germany (2003), Ireland (2013) and Italy (2014) [9,10]. Ordinances to eliminate clinical cases from the food chain were legislated in Austria and in Switzerland [9]. In one German region, a compulsory control program has been in force since 2017 [11]. In Thuringia, a federal state of Germany, a voluntary control program was firstly established in2003 and revised in 2008 and 2015 [12,13]. The evaluation report of the program phase from 2008 to 2014 revealed a limited participation rate [12], which is typical for most voluntary control programs, where voluntary continuation of the program among producers is much lower than hoped for [9]. Even for the Irish Johne’s Control Program, which is one of the most ambitious and well-managed programs in Europe, diminishing participation for both the completion of annual requirement by registered herds and recruiting new herds was observed; about 18% of the dairy cows in Ireland are enrolled [14].

Based on the Regulation (EU) 2016/429 of the European Parliament and of the Council of 9 March 2016 on transmissible animal diseases and amending and repealing certain acts in the area of animal health (‘Animal Health Law’), JD was a listed and, according to article 18, a notifiable disease in the European Union and a need for surveillance within the European Union was stated [15].

The Thuringian program has three objectives: reduce the level of infection when present; reduce the spread of Johne’s Disease to uninfected herds; and facilitate certification and protection of herds that are non-suspect with respect to JD [12,13]. In affected herds, the program focuses on prevalence reduction by hygienic measures to break the infection cycle, annual testing for the detection of animals shedding the infectious agent and culling of these animals. A certification pathway was established. Herds were certified as ‘non-suspect’ (NS) with respect to JD if the herd tested negative for three years. In order to establish adequate hygiene improvement in a farm-specific manner, the key elements are an initial veterinary risk assessment by specialized veterinarians of the Thuringian Animal Health Service and a control and management plan for each enrolled farm. The implementation of management and hygienic measures, such as trade control, calving hygiene, management of colostrum for the first feeding, feeding of unsaleable milk and personal hygiene in the calf and youngstock area, was enforced after being discussed with the farmer. Recommendations were given on the basis of scientific evidence [16] and considering the farmer’s needs and opportunities.

As JD is only one of several infectious diseases which are associated with lack of hygiene, measures that improve hygiene in order to prevent infections with MAP will likely also have positive effects on the occurrence of fecal-orally transmitted infections such as Rota- and Coronavirus, *Cryptosporidium parvum* or *Escherichia coli*. Often these infectious agents cause more severe clinical signs and health risks in calves than JD and may enhance farmers’ willingness to improve suboptimal hygiene [17]. A positive association between the occurrence of JD and the number of calves with diarrhea was shown in a study in Ontario dairy herds [18]. In addition, the Irish Johne’s Control Program has, besides others, the objective to improve calf health and farm biosecurity [18]. The annual calf mortality rate is a widely accepted indicator for calf health [19].

The aim of this case study was (1) to evaluate the progress of the Thuringian JD control program during the phase from 2015 to 2020 and (2) to investigate a possible association between the annual calf mortality rate and JD herd status within the Thuringian JD control program. We hypothesized that the annual calf mortality rate was associated with JD herd status.

## 2. Materials and Methods

### 2.1. Progress of the Thuringian JD Control Program

#### 2.1.1. Population

At the end of the reference period (2015–2020), the cattle population in Thuringia consisted of 293,862 cattle with 91,041 dairy cows and 37,644 cows in beef suckler cow-calf herds. About 84% of the dairy cows were pedigree bred, mostly of the German Holstein breed with a minor proportion of Simmental, Jersey and Brown Swiss [20]. During these years, the population downsized by about 20% dairy cows and 4% of cows in beef cow-calf suckler herds [20]. This development was contrary to those described for other European countries like Ireland [14]. The average 305-day milk yield of the tested German Holstein dairy cows in Thuringia constituted 10,153 kg in 2020 [20].

Dairy farms in Thuringia were mainly cooperative farms. Large dairy herds with more than 500 cows are typical for the dairy industry in Thuringia. About 60% of the cows were kept in those herds. Herds with ≤200 cows represent 10% of the population, and herds with >200–500 cows represent about 30% of the population [20]. In most operations, milk production was unseasonal, and pasturing of dairy cows was not very common. Therefore, calvings were nearly equally distributed around the year in most herds. Separate calving units or at least calving pens were common standards, in some cases operated by separate personnel. Most of the male calves were sold when at least two weeks old, and heifers were reared either at the owners’ farm or in cooperation with other dairy farmers in specialized operations situated in the more mountainous areas of Thuringia.

Beef herds were mainly operated in a suckler cow-calf system, with an average herd size of 16 cows. Simmental was the most popular breed. Disregarding several large herds, hobby or part-time farmers operate the majority of beef herds. A relevant percentage was engaged in pedigree breeding with a long tradition for beef-type Simmental breeding.

Until 1990, nearly no cases of JD were registered in Thuringia. The ‘iron curtain’ prohibited not only free travel for inhabitants but free animal trading as well. Although JD was widely spread in the (north-)western part of Germany, similar to all regions with a well-developed dairy sector, the eastern part of Germany (former German Democratic Republic) was well protected from the introduction of MAP. Cattle breeding was completely organized by state-owned operations in a way that genetics from abroad were imported only by bovine semen for artificial insemination and transferable embryos. The import of livestock by private farmers or cooperative farms was essentially prohibited. Triggered by a great market for Holstein cattle after the German reunification, a massive buy-in of livestock pushed the spread of JD into and within cattle herds in Thuringia and the other eastern federal states of Germany. The first clinical cases were confirmed several years later, during the late 1990s of the last century. Recently, a between-herd prevalence of about 50% was estimated for Thuringia as well as the whole eastern part of Germany [21,22].

#### 2.1.2. The Thuringian Johne’s Disease Control Program

The Thuringian JD Control Program is a collaboration between the Thuringian Ministry of Labor, Welfare, Health, Women and Family as the supreme veterinary authority in Thuringia, and the Thuringian Animal Health Service. This service is operated by the Thuringian Animal Disease Fund, a self-help organization of livestock farmers in Thuringia. Its original task is ensuring financial aid for animal disease control. The Ministry passed the program after consulting the Thuringian Farmers’ Association and Thuringian Cattle Breeders’ Association. The program is managed by the Thuringian Animal Health Service.

After an initial program phase during the years 2003–2007, which was based on semiannual serological whole-herd testing and general rules for improving hygiene management, the Thuringian JD control program has been comprised of the following principles since 2008:

1. On-farm veterinary risk assessment and tailored recommendations for improving hygiene management in order to break the infectious cycle between cows and calves;
2. Identification and removal of MAP shedders by annual testing of fecal samples from all cows for MAP to reduce the infective pressure;
3. Controlled buy-in of cattle in consideration of JD status of the herd of origin compared to the status of the own herd;
4. Certification of herds as ‘non-suspect’ regarding JD and maintenance of status by testing every two years.

The update of the control program in 2015 implemented the recommendations of the German Federal Ministry of Food and Agriculture that established different levels of control according to the diagnostic method used. In its present form, the program includes a control stage for MAP-positive herds, a certification stage for MAP-negative herds leading to certification with the status ‘non suspect’ regarding JD, and measures to maintain this status. The achievement of this status is described below. Due to the limited diagnostic sensitivity of animal-level tests [23,24], this status reflects a very low risk that the infectious agent was present in the herd. This risk is not zero but as low as MAP cannot be detected by repeated testing of the herd’s animals. This approach is common practice in animal disease control. The control stage was offered in three levels (1–3) as set by the federal recommendations. In addition, the Thuringian control program offers level 4 for farmers who aim at achieving the status ‘non-suspect’. In addition to improving hygiene management and testing of cattle showing clinical symptoms of JD, the following requirements are expected of farmers:

– Level 1: improve hygiene management and monitoring at the herd level by semiannual environmental sampling;
– Level 2: test and manage high-risk cows detected by annual individual testing for MAP-specific antibodies;
– Level 3: test and manage or remove MAP shedders as detected by annual testing of fecal samples from each cow for MAP;
– Level 4: test and remove MAP shedders in a timely fashion (special agreement, see below).

At enrollment, participating farmers can choose between levels 1 and 3 with respect to their individual goals and opportunities. Testing according to level 3 with negative results was a prerequisite to enter the certification pathway. Herds will enter the ‘certification phase’ if no MAP shedder has been identified in the respective year. A herd that had been tested accordingly with only negative results during three consecutive years will be certified as ‘non-suspect’. These herds were retested every two years for maintenance of the status.

For MAP-positive herds having a low prevalence of MAP shedders (<3%) and striving for the status ‘non-suspect’, the program offers a special agreement that binds farmers to remove MAP shedders from the herd within one month after detection (level 4). In level 4, exceptions were only accepted for pregnant cows in dairy herds and suckler cows. The general recommendation for MAP-positive dairy cows was that calves had to be removed from the cow before the first feed, and the cows had to be removed from the herd within one month after calving. A MAP-positive suckler cow can remain on pasture for more than one month to ensure the feeding, health and welfare of their calf. In ideal circumstances, this cow, together with its calf, should be separated from the rest of the herd and not re-bred. The calf was not intended for breeding, and the cow had to be slaughtered when the herd was taken off pasture, at the latest after one month off pasture. A financial incentive from the Animal Disease Fund was offered for all animals that were removed according to the specific rules of that level. This level demands a written commitment of the farmer because these management measures required in-detail advice, and this goal required high aspiration from the farmer.

Unless prompt culling of MAP shedders was recommended for each level, farmers in levels 1–3 could manage detected MAP shedders according to their pregnancy status, milk yield and hygiene management within the farm. The hygienic measures should have focused on calving hygiene and management of the calving pen, management of colostrum for the first feeding, feeding of unsaleable milk to calves, and staff hygiene in calf and youngstock units. An on-farm veterinary risk assessment was undertaken collaboratively by specialized veterinarians from the Animal Health Service and the farmer to systematically review the biosecurity risks of JD within the herd using a purpose-made questionnaire that served as a data record and the basis of discussing the herd-specific measures. Farmers were requested to agree to farm-specific management choices to reduce the likelihood of the introduction and spread of MAP and to adopt these measures in daily work.

Farmers were advised to focus on the JD status of the herd of origin when purchasing animals. All buy-in cattle should originate from herds with the same or higher stage of control or should be certified as ‘non-suspect’.

The program had the support of all involved stakeholders, recognizing it as having the capability to increase animal health and welfare in the enrolled herds. The program provides funded support for several activities. Costs of the whole-herd testing were shared by the farmer and the Thuringian Animals Disease Fund. For individual fecal sampling by a veterinarian, sampling assistance at the rate of EUR 2.00 per sampled animal was paid. For laboratory testing of the fecal samples, 50% of the testing costs were covered. For serological testing, testing assistance was provided at the rate of EUR 1.00 per tested animal and year. The Ministry paid the Animals Health Service to perform the veterinary risk assessment and counsel the farmers; additional expenses for improving farm hygiene were borne by the herd owner. There was no compensation for culling test-positive animals except for herds that joined level 4 by special agreement.

#### 2.1.3. Sampling and Testing

The Thuringian State Office for Consumer Protection, which is the competent authority for research ethics approval in Thuringia, approved the sampling within the control program and declared an exclusion from approval by an ethics committee (2684-04-15-TSK-22-201).

All tests regarding paratuberculosis were performed in the laboratory of the Thuringian Animal Health Service, which is accredited to DIN EN ISO/IEC 17025 for relevant tests.

All results of the laboratory tests were reported as soon as possible to the farmer and his veterinarian in written form (e-mail, fax, written report) and were electronically stored in the laboratory database of the Animal Health Service. Positive test results of fecal culture were reported to the competent veterinary authority as well because the detection of the infectious agent of paratuberculosis is notable in Germany.

##### 2.1.3.1. Sampling and Testing of Environmental Samples

In level 1-herds, environmental samples were collected either by the farmer or the herd’s veterinarian using either conventional environmental fecal sampling as described [25,26] or boot swabs and slurry sampling as used in previous studies [25,27]. Environmental fecal samples were collected by sampling 3–5 locations in manure accumulation areas, high cow-traffic areas and manure storage facilities [26]. Boot-swabs were collected by walking about 100–200 steps within the barn, preferably in fresh cow pens, main cow alleyways and the waiting pen in front of the parlour. Immediately after walking, boot swabs were placed in an aseptic plastic bag. Slurries were mixed before submerging a sampling container in the liquid manure storage facility (usually tanks, lagoons, pits or pre-flooders) to collect a sample of 150 mL slurry [27,28]. Environmental fecal samples and liquid manure samples were analyzed in an analogous manner to individual fecal samples (see Section 2.1.3.4 and Section 2.1.3.5), and boot swabs were preprocessed as described before [28].

##### 2.1.3.2. Sampling and Testing of Blood Samples

In level 2-herds, whole-blood samples were taken by the herds’ veterinarians using an EDTA-Kabevette (Primavette V EDTA, Kabe Labortechnik GmbH, Nümbrecht-Elsenroth, D) as provided by the laboratory. In most cases, aliquots of blood samples collected annually for animal disease monitoring (infectious bovine rhinotracheitis, brucellosis and others) were used. These samples were provided by the Thuringian state laboratory, which was responsible for the animal disease monitoring.

Blood samples were centrifuged for 5 min at 840× *g* (Rotanta TR 440, Hettich, Tuttlingen, D) before being analyzed for MAP-specific antibodies using a commercial ELISA test kit accredited for MAP diagnostics in Germany by the FLI (ID Screen Paratuberculosis Indirect ELISA kit, ID Vet, Montpellier, F). ELISA tests were accomplished according to the manufacturers’ instructions with the use of cut-offs as recommended by the manufacturer.

##### 2.1.3.3. Fecal Sampling

A fecal sample had to be taken from every cow to be tested in the framework of the control program, either to confirm clinical cases in level 1 and 2 herds or to perform the annual whole-herd tests in level 3 and 4 herds. Veterinarians were advised to take the samples rectally with an unused examination glove and wipe the feces off into a sterile plastic container supplied by the laboratory and equipped with a bar code. The samples were sent to the laboratory by a courier system provided by the Thuringian state laboratory within 24 to 48 h. The individual fecal samples were tested either by fecal culture or a commercial real-time PCR for the direct detection of MAP without a cultivation step. Because of comparable test performance, the test was chosen according to practical requirements. PCR was preferred in control stage farms for managing detected shedders because of the much quicker turnaround time, whereas for surveillance of ‘non-suspect’ farms, the fecal culture was preferred.

##### 2.1.3.4. Testing of Fecal Samples by Bacteriological Cultivation

Bacteriological cultivation was performed according to the official guidelines of diagnostic procedures published by the Friedrich–Loeffler Institut (FLI), the German Federal Research Institute for Animal Health [29] as described previously [28]. Herrold’s egg yolk medium (HEYM, Becton Dickinson, Heidelberg, Germany) was used as the cultivation medium. Characteristic colonies were confirmed by a conventional IS900 PCR [30]. Only samples with typical colonies without undesired overgrowth (usually mold) and a positive IS900 PCR result were characterized as MAP-culture positive.

##### 2.1.3.5. Testing of Fecal Samples by Real-Time PCR

For direct real-time PCR testing, we used the commercial bactotype MAP PCR kit (Indical Bioscience GmbH, Leipzig, Germany) after DNA extraction as recommended by the manufacturer. Samples with a Ct value less than or equal to 38 were considered MAP positive. Samples with a Ct value of 39–40 were considered inconclusive, and samples with no Ct value (>40) were considered negative.

### 2.2. Association between the Annual Calf Mortality Rate and JD Herd Status

#### 2.2.1. Study Design

In addition to the JD control program, some farms participated in a project regarding calf health performed by the Thuringian Animal Health Service (Calf Health Project) as described elsewhere [31]. Study herds were recruited among farms that belonged to the clientele of the Thuringian Animal Health Service participating in the Animal health program of the organization. Farmers were invited to take part in the study during December 2016 and January 2017 on a voluntary basis. A total of 93 dairy farms that agreed to participate were recruited. The mean herd size was 550 cows (min: 50, max: 1700). Cows of all herds were of the German Holstein breed. In all herds, cows were housed in free stalls. In the Calf Health Project, data on calf mortality of farms being enrolled in JD control program (*n* = 39) and farms being not enrolled (*n* = 54) were gathered as described below. Enrollment in the animal health program included the permission of the farmer to retrieve the data from the database.

#### 2.2.2. Data on Calf Mortality

Data on calf mortality of 93 dairy farms included in this analysis were retrieved from the German database on animal identification and registration (HI-Tier) [32]. German cattle farmers are obligated to report all cattle born in the herd and introduced to or removed from the herd to that database. The reason for their removal (e.g., sold, died, slaughtered) was to be reported as well. The retrieval encompassed the number of births given to calves within the year 2017 (denominator) and the number of calves that died before they were 6 months old in 2017 (numerator). The retrieval did not include stillborn calves, as they were not included in both the numerator and the denominator. These data on calf mortality exist for the years 2000 to 2017 for all 93 dairy farms. For further analysis, we considered calf mortality data of 10 consecutive years from the year before enrollment to 8 years after enrollment. In the case of enrollment and testing of individual fecal samples for MAP before 2009, we included the years starting with the year before this time-point. Calf mortality of the not enrolled farms was analyzed for the years 2008–2017.

#### 2.2.3. JD Herd Status Definition Based on Laboratory Testing

The JD status of the control program farms that were included in the analysis regarding the association between the annual calf mortality rate and JD herd status was defined according to the requirements of the control program as described in Section 2.2. The status ‘non-suspect’ required individual testing of fecal samples from each cow for MAP with only negative results during three consecutive years. A total of 13 farms out of the 93 Calf Health Project farms fulfilled these requirements. The other 26 farms were classified as control stage farms. The control program farms had to be enrolled since 2009 or earlier, ensuring individual testing of fecal samples for MAP of over 10 consecutive years. Laboratory testing was performed as described in Section 2.1.3.

#### 2.2.4. Statistical Analysis

Statistical analysis regarding calf mortality was performed using Microsoft Excel, version 2010 [33] and R software, version 4.1.2 (1 November 2021) [34] using R-package lme4 version 1.1-27.1. Calf mortality was not normally distributed and, therefore, was log-transformed for analysis purposes. Generalized linear mixed-effects models with hierarchically structured random effects (farm) using the maximum likelihood method were fitted to the data to identify associations between calf mortality outcome, years of enrollment in the control program and status of paratuberculosis (certified as ‘non-suspect’, herds in control stage or no enrollment). Estimated regression coefficients with *p* ≤ 0.05 were considered significantly different from zero.

## 3. Results

### 3.1. Progress of the Thuringain Johne’s Disease Control Programm

At the end of 2020, there were 163 cattle farms registered in the Thuringian JD control program. With respect to production type, there were 83 dairy farms with 38,842 dairy cows representing 43% of the dairy cow population and 89 beef herds with 7908 cows representing 21% of cows in beef cow-calf suckler herds. Some farms kept more than one dairy herd or a dairy and a beef herd.

During the reference period, a mean number of 25,320 individual fecal samples per year were tested either by fecal culture or by direct real-time PCR; the latter test has been available for routine diagnosis since 2017. The higher mean proportion of MAP-positive samples tested by real-time PCR of 2.7% compared to the percentage of fecal culture-positive samples reflected its preferred use in control stage farms, whereas fecal culture was preferably applied for the surveillance of ‘non-suspect’ herds (Table 1).

Only a few farms had chosen level 2 of the control stage, which refers to managing high-risk cows detected by annual individual testing for MAP-specific antibodies. A mean number of 2855 blood samples were tested for MAP-specific antibodies with 3.0% positive test results and a decreasing tendency from 2015 to 2020 (Table 1).

**Figure 1:**
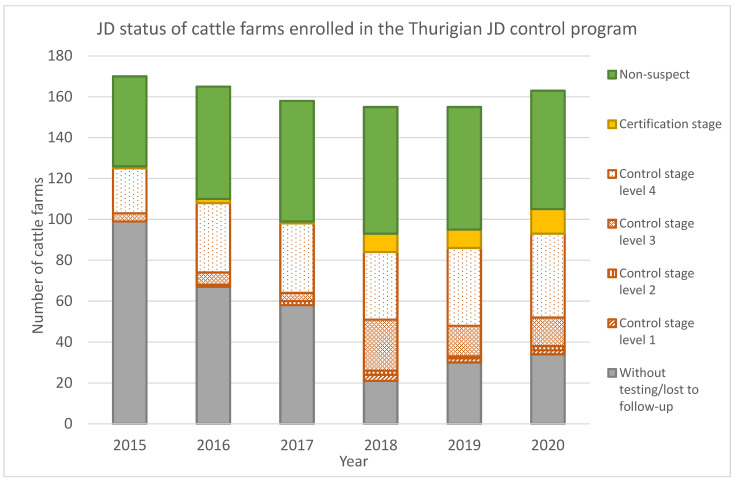
Number of cattle farms enrolled in the Thuringian Johne’s Disease control program with respect to the status achieved during the years 2015–2020.

By the end of 2020, 58 farms (35%) with 18,013 cows (14% of the population) were certified as ‘non-suspect’ with regard to JD, and 12 farms were in the certification stage (Figure 1). Prevalence reduction in control stage herds below the threshold of 3% MAP shedders resulted in an increasing proportion of farms in level 4 of the control stage compared to 2015. In 2015, a high proportion (99 farms) of herds were registered that did not perform the required annual whole-herd testing or that tested only a minor part of the herd. During the following 3 years, this flaw was reduced to 21 herds without testing or with insufficient testing. Due to economic reasons or testing fatigue, some farmers decided to omit further testing during the ensuing period, and the number of farms in this category increased again. Consequently, some farms were lost to follow-up. Testing fatigue was noticed in non-suspect herds as well. Testing the whole cow herd every two years only to maintain the status of ‘non-suspect’ with repeatedly completely negative results frustrated several herd owners, particularly those who could not benefit from the status when selling animals. This effect was reinforced during the COVID-19 pandemic, during which herd owners went without any public sales or shows.

**Figure 2:**
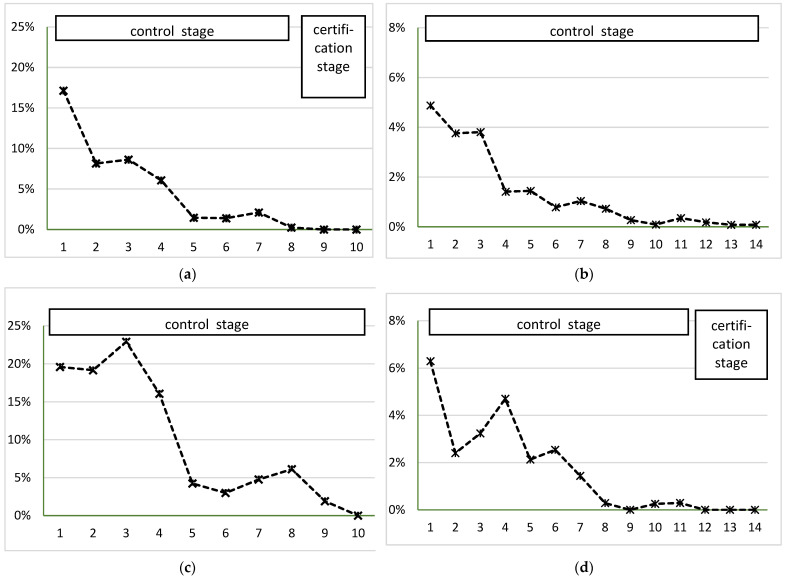
Prevalence of MAP shedders in 4 dairy herds enrolled in the Thuringian Johne’s Disease control program during 10 or 14 consecutive years of enrollment. (**a**) A herd with 420 dairy cows. Certification stage achieved after 8 years in the control stage. (**b**) A herd with 990 dairy cows. Long tailing-out period, with only one MAP shedder detected in years 10, 13 and 14 of enrollment. (**c**) A herd with 300 dairy cows. High initial prevalence, but no MAP shedders after 8 years in control stage. (**d**) A herd with 350 dairy cows. No MAP shedders after 8 years in control stage; long tailing-out period, with only one MAP shedder detected in years 10 and 11 of enrollment, respectively. Certification stage competed after 14 years and certified as ‘non-suspect’ in 2021.

The progress of Johne’s Disease control in the MAP-positive herds was demonstrated in Figure 2 by using the example of four typical Thuringian dairy herds. Reduction of MAP shedder’s within-herd prevalence during the control stage was shown for each of the four herds. In these farms, whole-herd testing, as recommended, was implemented immediately after enrollment. A modified testing schedule was used in herd (c), where cows were sampled during the 3rd or 4th week after calving to ensure reporting of test results before cows were re-bred. Identified MAP shedders were flagged (ear tag or nec band) and removed as soon as possible. Herd (a) removed heavy shedders immediately, did not re-breed low shedders and bred offspring of shedders to a terminal sire. In all four herds, calves were removed from dams without delay after calving and kept outside the barn in a separate unit (herd a) or igloos (herd b, c, d), and heifer calves were fed with colostrum and milk from negatively tested cows. Unsaleable milk from MAP shedders was not fed to heifer calves but either poured away or pasteurized (at least 30 min at 60 °C). In herd (b), individual calving pens and a separate calving pen for MAP shedders were available, but they were cleaned and disinfected only every two weeks and not after each calving.

### 3.2. Association between Calf Mortality and Johne’s Disease Herd Status

Overall, 834 observations of 93 dairy farms were included in the data analysis. JD status was significantly associated with calf mortality, such that farms staying in the control stage as well as farms not enrolled in the program had significantly higher values for calf mortality compared with farms certified as ‘non-suspect’ (Table 2). All fixed effects were tested for correlations to exclude multicollinearity, and none were found.

**Figure 3:**
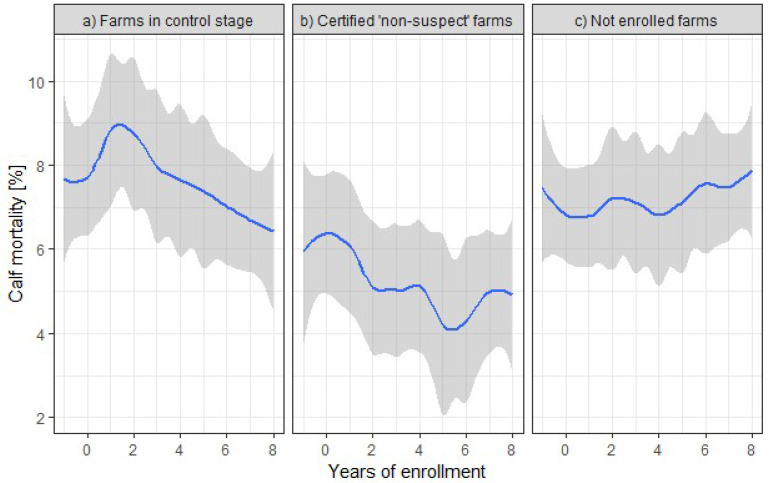
Mean (blue line) and 95% confidence interval (grey) of calf mortality during 10 consecutive years from the year before enrollment to 8 years after enrollment in Thuringian Johne’s Disease Control Program for dairy farms in the control stage (**a**), dairy farms certified as ‘non-suspect’ regarding JD during this period of time (**b**), and for dairy farms not being enrolled (observational period 2008–2017 (**c**).

In both groups of farms enrolled in the control program, values for calf mortality decreased over time (Figure 3). Initial calf mortality seemed to be lower in farms certified as non-suspect than in farms not reaching that stage. In detail, non-suspect farms decreased calf mortality on average from 5.87% to 4.21% in the 5th year after enrollment, and farms staying in the control stage decreased calf mortality on average from 7.85 to 6.45% in the 8th year. The calf mortality of unenrolled dairy farms remained at an average level of 7.19% within the 10 considered years, which compared well with the mean of calf mortality of all farms from 2000 to 2017 of 7.35% (95% confidence interval: 3.77–9.52%, minimum: 0%, maximum 47.7%).

## 4. Discussion

This case study describes the progress of a control program for JD in Thuringia during the period 2015–2020, continuing the program review of the period 2008–2014 [12]. Some issues that arose during the progress of the program and ways in which these could be managed as the program continues were addressed.

The four objectives established for the Thuringian JD control program for the period 2015–2020 were to:

1. Enhance the number of farms that achieved the status ‘non-suspect’ with regard to JD;
2. Reduce within-herd prevalence of MAP shedders in MAP-positive herds during the control stage by 50%;
3. Advance control in registered herds to classify at least 75% of enrolled herds in level 3 or 4 of the control stage, certification stage or certified as ‘non-suspect’;
4. Attain an enrollment rate of 50% of Thuringian dairy and beef cows in the program.

Compared to the period 2008–2014, progress was made regarding the certification of farms as ‘non-suspect’. Overall, 70 farms reached that goal or at least the certification stage, and among them were several herds with an initial prevalence of MAP shedders above 15% (Figure 2a,c). Due to the limited sensitivity of the test applied to the individual fecal samples (~70% for fecal culture or direct fecal real-time PCR, respectively) and the intermittent shedding in the earlier stage of the disease, the tests were not able to detect all MAP shedders. Therefore, an apparent within-herd prevalence of zero as measured with the applied tests will not justify the complete eradication of the infectious agent. Regardless of these limitations, the achieved sensitivity to detect a design prevalence of 1% was estimated to be 99% if all cows of a 400-cow dairy herd were tested with negative results, assuming a test specificity of 100% [35]. By testing multiple times with only negative results, herd-level sensitivity increases. Therefore, the performance of this surveillance system was accepted in Thuringia to certify farms as ‘non-suspect’ regarding JD if their cattle herds were tested three times with negative results as described. This ensures an acceptable confidence of freedom from disease, a sufficiently low risk of the presence of MAP in the respective herd and prevents further spreading of JD to other herds. In other words, 1 out of 7 Thuringian cows was kept in a ‘non-suspect’ herd and could be handled with a very low risk of spreading JD in 2020. This can be assumed to be a starting point of effective JD control in this region, where nearly half of the dairy herds were affected by JD [22]. Furthermore, the results of the Thuringian JD control program verify that the prevalence of MAP shedders can be reduced to a level where MAP is not detectable anymore and that it is possible to keep this status for at least three years. The requirements to certify a farm as ‘non-suspect’ were in line with the federal recommendations and applied in other federal states as well, e.g., Saxony and Mecklenburg-West Pomerania. Furthermore, the proof of the absence of MAP shedders over a period of three consecutive years fulfills similar requirements for certification as in other programs, e.g., the Uniform Program Standards for the Voluntary Bovine Johne’s Disease Control Program in the US [36].

By end of 2020, 43% of Thuringian dairy cows were enrolled in the program. This was in line with the level of participation achieved in other voluntary JD control programs, such as those in Ireland, Italy or Denmark [14,37,38]. Nonetheless, diminishing participation, particularly for recruiting new herds, is a current challenge. The ambitious goal to involve half of the cows kept in Thuringia by 2020 was not attained. Despite the fact that the costs of the program for participating farmers were limited (half of the testing costs and some additional efforts to improve hygiene) and were low compared to the benefits, the majority of Thuringian cattle farmers have yet to register in the program. This reticence was consistent with our findings in a previous study regarding attitudes towards paratuberculosis control using cluster analysis applied on data from an anonymous survey among cattle farmers in Thuringia and Saxony [39]. In this study, we identified four groups of farmers tagged ‘free supporters’, ‘affected supporters’, ‘sceptics’, and ‘deniers’. In contrast to the supporters of the program, the two groups of farmers tagged as ‘sceptics’ and ‘deniers’ did not consider paratuberculosis a dangerous epizootic disease, and the ‘deniers’ would not enroll in a voluntary control program even if the pathogen would have been found in their herd. These farmers mentioned the costs and the inaccuracy of diagnostic tests as obstacles that hamper their enrollment [39]. The reluctance of farmers to adopt JD control measures was often justified with the argument that existing tests fall short of offering a reliable basis for consistent control and that the available tests still do not offer the necessary sensitivity and specificity. The Thuringian JD control program clearly demonstrates that it was possible to take control of the disease even with the existing tests.

An Irish study revealed that farmers’ decisions on whether to introduce control and preventive measures are not solely influenced by economic consequences. Rather the value that farmers associate with social relationships with others and with particular objectives and actions informed farmers’ decisions [40]. Other studies highlight factors such as discussion amongst peers, perception of responsibility, confidence in professional advice, problem awareness, effectiveness of recommended strategies and farmers’ ability to implement recommended management practices rather than perceived risk or perceived benefits and disadvantages as factors that influence farmers’ decisions [41,42]. Unless these studies pointed out the need for tailored communication strategies, identifying the appropriate way of communication for each farmer was challenging in practice. In Thuringia, the structure of the dairy sector with its small number of large herds allows repeated individual counselling of the decision makers by specialized veterinarians of the Animal Health Service. Considering this rather individual communication strategy in the light of the achieved participation reveals the limitations of communication to convince farmers to voluntarily implement preventive measures for infectious disease control. From the authors’ perspective, increasing social pressure resulting from a high uptake of the program by the majority of cattle owners, including the pedigree breeders, may have the potential to increase the uptake of the program. Alternatively, mandatory active surveillance based on either milk screening for MAP-specific antibodies or testing environmental samples for MAP would increase the perception of JD among farmers, as observed in Lower Saxony. In 2017, mandatory surveillance based on either individual or pooled test day milk samples was enacted in that federal state, and the uptake of the voluntary control program increased remarkably [43]. This example represents the two-stage approach of JD control with mandatory surveillance in a region and tailored voluntary control measures at the herd level, as suggested before [44]. Another successful example of two-stage JD control is the voluntary program in Tyrolian cattle herds that involved more than 4679 farms comprising 70% of the cattle population in the first run. This monitoring based on boot swab testing was combined with voluntary control measures in 131 MAP-positive herds [25].

A reduction in the prevalence of MAP shedders was achieved in most of the participating herds. In most herds, prevalence reduced remarkably within the first five years after enrollment, as shown by the examples in Figure 2a,c. The aspect of prevalence reduction was described in detail in the program review for the years 2008–2014 [12] and was in line with the results of other studies [45,46]. After the initial decrease of MAP shedders’ prevalence, we observed a slowing down in the reduction of the prevalence (e.g., Figure 2b,d) and a long tailing-out period. When it comes to prevalence-based data, it is important to keep in mind that prevalence does not completely reflect the progress of control within a herd. Firstly, because culling as part of the control measures directly affects prevalence, reduction of prevalence might reflect an apparent reduction of cases, but not an obligatory break of the infection cycle. Secondly, because of the long incubation period of JD, infected animals might not be detected and may appear as new cases later on. As an example, the increase in prevalence in herd (c) of Figure 2 during the first three years reflects this effect. Thirdly, if shedders were removed from the herd in a timely fashion as recommended, prevalence, as determined here, essentially includes only new cases. In the Thuringian control program, farmers were advised not to retest MAP shedders that were identified in the preceding herd testing. Under these circumstances, the prevalence was an approximation of cumulative incidence (new cases), which reflects the new infections several years before. The reduction of the number of MAP shedders within the herd, be it by lowering the rate of new infections or targeted culling of the affected animals, was a pivotal element of the control progress because it leads to less contamination within the cattle barn and a lowered risk for susceptible animals to become infected. Furthermore, it lowers the risk of undetected MAP-infected animals transmitting the disease from one herd to another.

Improvements in hygiene standards may result in a reduction in prevalence as well [47], assuming an optimum interruption of the infection chain. However, practical experience in JD control in Thuringia has shown that perfect hygiene management is hard to keep up in the long run. Therefore, reducing the infective pressure in the cattle barn environment by the detection and removal of MAP shedders before they reach the clinical stage was of great importance for successful control, as also shown by modelling [48].

Changes in the management and structure of the maternity area of a cattle operation leading to a relevant improvement of calving and calf-rearing hygiene will be valuable for reducing the burden of JD and other infectious calf diseases (e.g., neonatal calf diarrhea, respiratory disease). Besides omphalitis, diarrhea was the most common calf disorder in a recent German study and was most frequently found in dairy herds in the eastern part of Germany [49]. A link between the occurrence of JD and the number of calves with diarrhea was shown in Ontario dairy herds [18]. The economic benefit of JD control results not only from the reduction of the JD burden but also from the reduction of the prevalence of neonatal calf diarrhea [50]. The results of our study suggest for the first time that the status of a herd achieved in JD control program was associated with the annual calf mortality rate as an indicator of calf health within a dairy herd. The level of calf mortality in the non-enrolled dairy herds was in the range reported for Thuringian dairy herds for the years 2006–2007 (7.7%) [51] and for dairy herds with high calf mortality in northern Germany (7.1%) [52]. In the dairy herds that registered in the control program, a reduction of calf mortality was obvious, and for the ‘non-suspect’ herds, it was statistically significant. The strength of our finding is that the JD status of the enrolled dairy herds was well-defined according to the Thuringian JD control program, and it was unlikely that the non-enrolled farms had any alternative JD control in place. Furthermore, the results were based on a longitudinal study design with a clear intervention (namely JD control) which was more valid than a cross-sectional study design. Otherwise, there were several limitations of the study. On the one hand, the selection of the herds was not a random sampling, and progressive farmers with high aspirations regarding animal health may have been overrepresented. On the other hand, no other dairy herds were available that fulfilled the inclusion criteria, particularly regarding fecal testing for MAP during 10 consecutive years. For this reason, the number of involved herds, particularly in the group of certified herds, was small, limiting the power of the study. Furthermore, the knowledge of farmers regarding rearing and feeding of calves may have increased during a period of ten years, and newly built calf-rearing units may have facilitated calf health. Therefore, our findings have to be interpreted with caution. Future studies that combine research on calf health with JD monitoring should focus on this issue in depth.

Further development and continuation of the Thuringian JD Control program holds knowledge that may assist others running a JD control program. A permanent challenge was the continuation of the required testing schedule, particularly if the farm was certified as ‘non-suspect’ and testing was required to maintain this status. The owners of these farms demanded a remarkable reduction of the testing efforts. Ensuring a very low risk of having JD or high confidence of freedom from disease only by means of testing is hampered by the low sensitivity of easily available herd-level tests, such as repeated testing of pooled test day milk samples or bulk milk [53]. Data on repeated environmental sampling is rare [54], but recent modelling studies have shown promising results [55]. Testing environmental samples for MAP is an easy-to-use diagnostic approach ensuring a high specificity, but sensitivity depends on the within-herd prevalence of MAP-shedders [56] and valid sampling would be challenging. An evaluation study in Thuringian dairy herds is underway to elucidate the potential of repeated environmental sampling or pooled milk testing to establish confidence in freedom from JD at an acceptable level. From a more practical view, sophisticated testing, be it on the basis of sufficient individual samples or herd level samples at sufficiently frequent intervals, is always a challenge when it should be maintained in a voluntary setting. As data on animal movement are available at a very high quality for cattle in Germany, the involvement of movement data in combination with the JD status of the herd of origin in the calculation of a JD risk score might be advantageous and has the potential to reduce testing efforts, particularly for ‘non-suspect’ herds. Interestingly, the majority of owners of Map-positive herds in the control stage did not complain as much because of the long tailing out period during that stage. Nonetheless, a few MAP-positive test results after a period without a positive test result were dissatisfying. Some herd owners handled that problem by rather aggressive culling during the last years of the control stage, where they removed all cows that tested positively either for MAP-specific antibodies or MAP, including their offspring. Breeding the offspring to a terminal sire was an alternative approach, which was, regrettably, rarely used until now.

In the context of commercial cattle farming, be it dairy or beef, a high technical standard of the control program was expected, but pragmatism will be needed at several points to retain the acceptance of the program. The strategic engagement of key stakeholders in agreeing on program updates and sharing responsibility for decision-making was a critical point. The involvement of pedigree breeders in the dairy sector and their associations was a challenge because they were afraid of restriction for the export of breeding cattle into third countries if their herd was deemed MAP-positive by the veterinary authority. This was the case if MAP was detected by bacterial cultivation because the detection of the infectious agent is noticeable to competent authority according to the European Animal Health Law. This may hamper export certification for several countries outside the EU. Pedigree breeders in the beef sector were more willing to join the program as they were selling at a regional or national market, and an official veterinary certification for the national or EU market is not mandated in the case of JD. Moreover, the Animal Health Law in its present form ‘guarantees’ the absence of veterinary trading restrictions regarding JD within the EU. At the EU level, there were no requirements regarding JD, and national measures shall not hinder the movement of animals and products between Member States. Thus far, when trading animals, only the buyer can prevent the introduction of MAP into his herd by infected animals in the subclinical stage of the disease.

Future development of the Thuringian JD control program requires the improvement of communication strategies to reach the less innovative and ‘late-adopting’ herd owners. Some of them might be willing to register in the program, but they were sceptic regarding the benefit for their herd. A different communication strategy may be required to convince this group. Informative websites and social media channels may be helpful and should be developed in the near future. Changing the mind of ‘deniers’ would not succeed only by information campaigns. A mandatory surveillance system as established in Lower Saxony is expected to be more effective to motivate JD control in that group. Results of that monitoring, together with movement data, should inform an easy-to-use herd scoring system that provides information regarding the JD status of the respective herd for potential buyers of cattle. Ideally, such a system should be established as a market assurance program that is open for all cattle owners and not only for those who were enrolled in a voluntary control program. This would significantly reduce the risk of buy-in MAP together with cattle from other herds and limit the spread of JD between herds.

## 5. Conclusions

We conclude that JD control was achievable at the herd level for years provided that veterinary advice, sufficient testing capacities and financial aid were available to the farmers and the re-introduction of MAP was prevented. Reduction of prevalence was feasible within several years, but reduction to a level where MAP was not detectable anymore takes a period of 10–15 years. Aiming at certification as ‘non-suspect’ regarding JD requires high aspiration and commitment from the farmer but was the aspiration of a relevant proportion of Thuringian cattle farmers. The annual calf mortality rate was reduced in dairy herds certified as ‘non-suspect’, reflecting an improved level of hygiene and calf health. Participation in this voluntary control program was limited to the more innovative and progressive farmers if not accompanied by a mandatory surveillance system. The involvement of the beef sector was worthwhile, as demonstrated by the enrollment of beef farmers in the Thuringian JD control program. Further development of the control program is required, particularly regarding the surveillance of ‘non-suspect’ herds and to establish an easy-to-use risk scoring system reflecting the potential herd-level risk of JD for potential buyers of cattle.

## Figures and Tables

**Table 1:** Number of samples analyzed for *Mycobacterium avium* subspecies *paratuberculosis* (MAP) in the Thuringian JD control program during the years 2015–2020.

Year	2015	2016	2017	2018	2019	2020
Bacteriological cultivation of fecal samples						
*n* ^1^	25,587	25,557	18,360	17,010	17,777	16,696
MAP+ [%] ^2^	641 [2.5]	343 [1.3]	228 [1.2]	272 [1.6]	289 [1.6]	171 [1.0]
Real-time PCR applied to fecal samples						
*n* ^1^			6869	8993	5681	9390
MAP+ [%] ^2^			146 [2.1]	315 [3.5]	193 [3.4]	189 [2.0]
ELISA applied to blood samples						
*n* ^1^	3210	3135	3657	4078	3465	2437
MAP-Ab+ [%] ^3^	104 [3.2]	108 [3.4]	132 [3.6]	114 [2.8]	86 [2.5]	47 [1.9]

^1^ Number of samples analyzed, ^2^ number of samples positively tested for *Mycobacterium avium* subspecies *paratuberculosis* (MAP), percentages given in parentheses, ^3^ number of samples positively tested for *Mycobacterium avium* subspecies *paratuberculosis* specific antibodies (MAP-Ab), percentages given in parentheses.

**Table 2:** Results of the generalized linear mixed-effects model for variables associated with calf mortality.

Fixed Effects	Estimate	Standard Error	*p*-Value
Years of enrollment	−0.002	0.003	0.489
Farms certified as ‘non-suspect’ regarding JD (*n* = 13)	Reference		
Farms in control stage (*n* = 26)	0.229	0.088	0.0104
Farms not enrolled in program (*n* = 54)	0.172	0.083	0.0419

## Data Availability

Data is contained within the article (Table 1, Table 2 and Table A1. Details of laboratory analysis are available on request.

## Appendix A

**Table A1:** Purpose-made questionnaire used by the specialized veterinarians from the Animal Health Service to systematically review the biosecurity risks of JD within a dairy herd.

Question	Improvement (Green) ^1^	Acceptable Standard (Yellow) ^2^	Need of Improvement (Red) ^3^
Separation of calving area within the holding	Well separated from other cow pens	Close to dry and fresh cows, separated from lactating cows	No separation
Separate calving pen for MAP shedders	Yes	Temporary	No
Type of calving pen	Individual calving pen	Group pens of ≤ 3 cows or tie-up stalls	Group pens of > 3 cows
Littering of calving pen	Ridding the complete manure and new littering after each calving	New littering after each calving, dry bedding	Wet and manure-contaminated bedding
Board of calving pen	Clean	Minor manure contamination	Extensive manure contamination
Washing of calving pen	After each calving	At least once weekly	Seldom or never
Cow-calf separation after calving	Within one hour	1–4 h after calving	>4 h after calving
Cleaning of cows before calving	Routinely washing of cows	No washing, most cows are clean	No washing, most cows are dirty
Cleaning of neonates	Routinely washing and drying	Rubbing with straw	No cleaning of neonates
Obstetrical hygiene (obstetrician and instruments)	Cleaning and disinfection	Cleaning without disinfection	No cleaning nor disinfection
Hygiene of colostrum milking	Well cleaned udders, vacuum system	Well cleaned udders, milking by hand	Milking without cleaning or suckling
Source of colostrum for first feeding	Negatively tested cows, preferably own dam	Own dam if not a known MAP shedder	Any cow regardless of test results
Timespan between calving and first colostrum feeding	Within one hour after calving	1–4 h after calving	>4 h after calving
Colostrum stored in the colostrum bank	From bacteriologically MAP-negative cows	From serologically MAP-negative cows	From untested cows
Documentation of colostrum feeding	Permanently traceable for each calf	Traceable for calves given colostrum from other cows than their dam	No documentation
Cleaning of milk feeding utensils	After each feeding	Cleaning daily, use of individual buckets	Cleaning less frequent than daily
Use of milk from MAP-positive cows for calf feeding	No use	Use after pasteurization	Use without pasteurization
Cow manure on feeding utensils	No manure	Traces of manure, utensils regularly washed	Manure contamination
Location of calf pens within the barn	Single pen in an area separated from cows	Group pen in an area separated from cows	Together with cows
Cleaning and disinfection of calf pens	All-in-all-out with cleaning and disinfection in between	Mucking out daily, cleaning at least weekly	No cleaning
Cow manure in water buckets and feed bunks	Clean calf feed and water containers	Technologically minimized manure contamination	Manure contamination visible
Milk not suitable for sale fed to calves	Never	After pasteurization	Without pasteurization
Quality of roughage (hay, haylage, silage) for calves in first two months	From fields without application of liquid manure	Fresh roughage	Remnant cow feed
Separation of weaned heifer’s holding from cow’s holding	Separated site	Separate pens at the same site	Close proximity to cows
Cleaning and disinfection of weaned heifer’s pens	All-in-all-out, cleaning and disinfection	Mucking out after each occupancy without cleaning	Mucking out on demand
Staff hygiene in youngstock holdings	Separate staff	Changing of clothes and cleaning boots	No measures, contaminated with cow’s manure
Skid-steer usage in youngstock’s holding	Separate skid-steer	Cleaned from cow’s manure before usage	Use without cleaning from cow’s manure
Pasturing of weaned heifers younger than one year	Never	Only on pastures without cow pasturing during the last season	Heifers share pastures with cows

^1^ The green (left) column represented the management practice considered an improvement of the hygiene standard with respect to limiting the spread of MAP within the herd. ^2^ The response options in the yellow (standard) column represent the management practice considered an acceptable practice in Thuringian dairy herds. ^3^ The red (right) column described management practices that should be avoided when JD control is aimed for.

## References

[1] Whitlock R.H., Buergelt C. (1996). Preclinical and clinical manifestations of paratuberculosis (including pathology). Vet. Clin. North Am. Food Anim. Pract..

[2] Tiwari A., VanLeeuwen J.A., McKenna S.L., Keefe G.P., Barkema H.W. (2006). Johne’s disease in Canada: Part I: Clinical symptoms, pathophysiology, diagnosis, and prevalence in dairy herds. Can. Vet. J..

[3] McAloon C.G., Whyte P., More S.J., Green M.J., O’Grady L., Garcia A., Doherty M.L. (2016). The effect of paratuberculosis on milk yield—A systematic review and meta-analysis. J. Dairy Sci..

[4] Kudahl A.B., Nielsen S.S. (2009). Effect of paratuberculosis on slaughter weight and slaughter value of dairy cows. J. Dairy Sci..

[5] Garcia A.B., Shalloo L. (2015). Invited review: The economic impact and control of paratuberculosis in cattle. J. Dairy Sci..

[6] Ayele W.Y., Svastova P., Roubal P., Bartos M., Pavlik I. (2005). *Mycobacterium avium* subspecies *paratuberculosis* cultured from locally and commercially pasteurized cow’s milk in the Czech Republic. Appl. Environ. Microbiol..

[7] Waddell L.A., Rajić A., Stärk K.D., McEwen S.A. (2015). The zoonotic potential of *Mycobacterium avium* ssp. *paratuberculosis*: A systematic review and meta-analyses of the evidence. Epidemiol. Infect..

[8] Kuenstner L., Kuenstner J.T. (2021). *Mycobacterium avium* ssp. *paratuberculosis* in the Food Supply: A Public Health Issue. Front. Public Health.

[9] Whittington R., Donat K., Weber M.F., Kelton D., Nielsen S.S., Eisenberg S., Arrigoni N., Juste R., Sáez J.L., Dhand N. (2019). Control of paratuberculosis: Who, why and how. A review of 48 countries. BMC Vet. Res..

[10] Geraghty T., Graham D.A., Mullowney P., More S.J. (2014). A review of bovine Johne’s disease control activities in 6 endemically infected countries. Prev. Vet. Med..

[11] Krieger M., Eisenberg S., Köhler H., Freise F., Campe A. (2021). Within-herd prevalence threshold for the detection of *Mycobacterium avium* ssp. *paratuberculosis* antibody-positive dairy herds using pooled milk samples: A field study. J. Dairy Sci..

[12] Donat K. (2017). The Thuringian bovine paratuberculosis control programme-results and experiences. Berl. Munch. Tierarztl. Wochenschr..

[13] Thuringian Ministry for Labour, Welfare, Health, Woman and Family Thüringer Programm zur Bekämpfung der Paratuberkulose in den Rinderbeständen in Thüringen. https://www.tmasgff.de/fileadmin/user_upload/Veterinaerwesen/Dateien/Tiergesundheit/Landesprogramme_Tiergesundheit/programm_zur_bekampfung_der_paratuberkulose.pdf.

[14] Gavey L., Citer L., More S.J., Graham D. (2021). The Irish Johne’s Control Programme. Front. Vet. Sci..

[15] Regulation (EU) 2016/429 of the European Parliament and of the Council of 9 March 2016 on Transmissible Animal Diseases and Amending and Repealing Certain Acts in the Area of Animal Health (‘Animal Health Law’). https://eur-lex.europa.eu/legal-content/EN/TXT/PDF/?uri=CELEX:32016R0429&from=DE.

[16] Doré E., Paré J., Côté G., Buczinski S., Labrecque O., Roy J.P., Fecteau G. (2012). Risk factors associated with transmission of *Mycobacterium avium* subsp. *paratuberculosis* to calves within dairy herd: A systematic review. J. Vet. Intern. Med..

[17] Barkema H.W., Orsel K., Nielsen S.S., Koets A.P., Rutten V., Bannantine J.P., Keefe G.P., Kelton D.F., Wells S.J., Whittington R.J. (2018). Knowledge gaps that hamper prevention and control of Mycobacterium avium subspecies paratuberculosis infection. Transbound. Emerg. Dis..

[18] Sorge U.S., Lissemore K., Godkin A., Jansen J., Hendrick S., Wells S., Kelton D.F. (2012). Risk factors for herds to test positive for *Mycobacterium avium* ssp. *paratuberculosis*-antibodies with a commercial milk enzyme-linked immunosorbent assay (ELISA) in Ontario and western Canada. Can. Vet. J..

[19] Cuttance E., Laven R. (2019). Estimation of perinatal mortality in dairy calves: A review. Vet. J..

[20] Trends of Animal Breeding in Thuringia. Reporting year 2020. Report of the Thuringian State office for Agriculture and rural area. https://tlllr.thueringen.de/fileadmin/TLLLR/Service/Publikationen/Schriftenreihe/2021_TZB_2020.pdf.

[21] Pützschel R., Einax E., Zoche-Golob V., Donat K. (2017). Spread of infection with *Mycobacterium avium* ssp. *paratuberculosis* (MAP) in cattle herds in Saxony and Thuringia on herd level. Berl. Munch. Tierarztl. Wochenschr..

[22] Eisenberg S., Krieger M., Campe A., Lorenz I., Einax E., Donat K. (2022). Herd prevalence estimation of *Mycobacterium avium* Subspecies *paratuberculosis* burden in the three main dairy production regions of Germany (PraeMAP). Animals.

[23] Collins M.T., Gardner I.A., Garry F.B., Roussel A.J., Wells S.J. (2006). Consensus recommendations on diagnostic testing for the detection of paratuberculosis in cattle in the United States. J. Am. Vet. Med. Assoc..

[24] Donat K., Schlotter K., Erhardt G., Brandt H.R. (2014). Prevalence of paratuberculosis in cattle and control measures within the herd influence the performance of ELISA tests. Vet. Rec..

[25] Khol J.L., Eisenberg S., Noll I., Zschöck M., Eisenberg T., Donat K. (2019). Two-stage control of paratuberculosis: Herd-status surveillance as the basis for operational measures to reduce the prevalence. Experiences from Lower Saxony, Hesse, Thuringia and Tyrol. Tierarztl Prax Ausg G Grosstiere Nutztiere.

[26] Donat K., Schau U., Soschinka A. (2011). [Identification of *Mycobacterium avium* ssp. paratuberculosis infected dairy herds by environmental sampling]. Berl. Munch. Tierarztl. Wochenschr..

[27] Eisenberg T., Wolter W., Lenz M., Schlez K., Zschöck M. (2013). Boot swabs to collect environmental samples from common locations in dairy herds for Mycobacterium avium ssp. paratuberculosis (MAP) detection. J. Dairy Res..

[28] Hahn N., Failing K., Eisenberg T., Schlez K., Zschöck P.M., Donat K., Einax E., Köhler H. (2017). Evaluation of different diagnostic methods for the detection of *Mycobacterium avium* subsp. *paratuberculosis* in boot swabs and liquid manure samples. BMC Vet. Res..

[29] FLI Official Manual of Diagnostic Procedures. https://www.openagrar.de/rsc/viewer/openagrar_derivate_00028517/TK18-Paratuberkulose-2020-07-08.pdf?page=22.

[30] Englund S., Ballagi-Pordány A., Bölske G., Johansson K.E. (1999). Single PCR and nested PCR with a mimic molecule for detection of Mycobacterium avium subsp. paratuberculosis. Diagn. Microbiol. Infect. Dis..

[31] Immler M., Failing K., Gärtner T., Wehrend A., Donat K. (2021). Associations between the metabolic status of the cow and colostrum quality as determined by Brix refractometry. J. Dairy Sci..

[32] HI-Tier. https://www.hi-tier.de.

[33] Microsoft Corporation Development Team (2020). Microsoft Excel.

[34] (2020). R Core Team R: A Language and Environment for Statistical Computing.

[35] Ausvet 1-Stage Freedom Analysis. Sample Size for Freedom Surveys. https://epitools.ausvet.com.au/freedomss.

[36] Uniform Program Standards for the Voluntary Bovine Johne’s Disease Control Program. https://www.aphis.usda.gov/animal_health/animal_diseases/johnes/downloads/johnes-ups.pdf.

[37] Arrigoni N., Garbarino C., Boldini M., Ruocco L., Gemma Brenzoni L., Gradassi M., Leo S., Paternoster G., Tamba M. Bovine paratuberculosis in Italy: Results after the first two years of application of the national guidelines. Proceedings of the 5th ParaTB-Forum.

[38] Nielsen S.S., Krogh K. Developments in the Danish Control Program on Paratuberculosis. Proceedings of the 4th ParaTB-Forum.

[39] Zoche-Golob V., Pützschel R., Einax E., Donat K. (2021). Identification of different attitudes towards paratuberculosis control using cluster analysis applied on data from an anonymous survey among German cattle farmers. Ir. Vet. J..

[40] McAloon C.G., Macken-Walsh Á., Moran L., Whyte P., More S.J., O’Grady L., Doherty M.L. (2017). Johne’s disease in the eyes of Irish cattle farmers: A qualitative narrative research approach to understanding implications for disease management. Prev. Vet. Med..

[41] Roche S.M., Jones-Bitton A., Meehan M., Von Massow M., Kelton D.F. (2015). Evaluating the effect of Focus Farms on Ontario dairy producers’ knowledge, attitudes, and behavior toward control of Johne’s disease. J. Dairy Sci..

[42] Ritter C., Jansen J., Roth K., Kastelic J.P., Adams C.L., Barkema H.W. (2016). Dairy farmers’ perceptions toward the implementation of on-farm Johne’s disease prevention and control strategies. J. Dairy Sci..

[43] Donat K., Eisenberg S., Köhler H. Paratuberculosis in Germany—Next step forward to control in cattle herds. Proceedings of the 6th ParaTB-Forum.

[44] Donat K., Eisenberg S., Whittington R., Behr M.A., Stevenson K., Kapur V. (2020). Paratuberculosis Control Measures. Paratuberculosis Organism, Disease, Control.

[45] Collins M.T., Eggleston V., Manning E.J. (2010). Successful control of Johne’s disease in nine dairy herds: Results of a six-year field trial. J. Dairy Sci..

[46] Ferrouillet C., Wells S.J., Hartmann W.L., Godden S.M., Carrier J. (2009). Decrease of Johne’s disease prevalence and incidence in six Minnesota, USA, dairy cattle herds on a long-term management program. Prev. Vet. Med..

[47] Kudahl A.B., Nielsen S.S., Østergaard S. (2008). Economy, efficacy, and feasibility of a risk-based control program against paratuberculosis. J. Dairy Sci..

[48] Marcé C., Ezanno P., Seegers H., Pfeiffer D.U., Fourichon C. (2011). Predicting fadeout versus persistence of paratuberculosis in a dairy cattle herd for management and control purposes: A modelling study. Vet. Res..

[49] Dachrodt L., Arndt H., Bartel A., Kellermann L.M., Tautenhahn A., Volkmann M., Birnstiel K., Do Duc P., Hentzsch A., Jensen K.C. (2021). Prevalence of disorders in preweaned dairy calves from 731 dairies in Germany: A cross-sectional study. J. Dairy Sci..

[50] Roche S.M., Von Massow M., Renaud D., Shock D.A., Jones-Bitton A., Kelton D.F. (2020). Cost-benefit of implementing a participatory extension model for improving on-farm adoption of Johne’s disease control recommendations. J. Dairy Sci..

[51] Zitzmann R., Pfeffer M., Söllner-Donat S., Donat K. (2019). Risikofaktoren für die Kälbersterblichkeit beeinflussen den Nachweis von Antikörpern gegen die Erreger der enzootischen Bronchopneumonie [Risk factors for calf mortality influence the occurrence of antibodies against the pathogens of enzootic bronchopneumonia]. Tierarztl Prax Ausg G Grosstiere Nutztiere.

[52] Tautenhahn A., Merle R., Müller K.E. (2020). Factors associated with calf mortality and poor growth of dairy heifer calves in northeast Germany. Prev. Vet. Med..

[53] Meyer A., McAloon C.G., Tratalos J.A., More S.J., Citer L.R., Graham D.A., Sergeant E. (2019). Modeling of alternative testing strategies to demonstrate freedom from Mycobacterium avium ssp. paratuberculosis infection in test-negative dairy herds in the Republic of Ireland. J. Dairy Sci..

[54] Sergeant E., McAloon C.G., Tratalos J.A., Citer L.R., Graham D.A., More S.J. (2019). Evaluation of national surveillance methods for detection of Irish dairy herds infected with Mycobacterium avium ssp. paratuberculosis. J. Dairy Sci..

[55] Zoche-Golob V., Donat K., Barkema H.W., De Buck J., Kastelic J., Wolf R. (2021). Predicting sensitivity of repeated environmental sampling for Mycobacterium avium subsp. paratuberculosis in dairy herds using a Bayesian latent class model. Vet. J..

[56] Donat K., Kube J., Dressel J., Einax E., Pfeffer M., Failing K. (2015). Detection of Mycobacterium avium subspecies paratuberculosis in environmental samples by faecal culture and real-time PCR in relation to apparent within-herd prevalence as determined by individual faecal culture. Epidemiol. Infect..

